# Editorial: Targeting Heterogeneity of Mesenchymal Stem Cells

**DOI:** 10.3389/fcell.2022.894008

**Published:** 2022-04-06

**Authors:** Qi Zhang, Yan Xu, Jianyong Xu

**Affiliations:** ^1^ Biotherapy Centre, The Third Affiliated Hospital, Sun Yat-sen University, Guangzhou, China; ^2^ Department of Immunology, School of Medicine, Health Science Center, Shenzhen University, Shenzhen, China

**Keywords:** mesenchymal stem cells, MSCs, heterogeneity, lipidomics, iMSCs, Dpscs, single-cell RNA sequencing, PDLSCs

Mesenchymal stem cells (MSCs) are multipotent cells with immune-modulatory properties and have great potential for cell therapy of various diseases (Wang et al., 2014; Xu, 2018; Jiang and Xu, 2020; Levy et al., 2020). MSCs could be isolated from various tissues, such as bone marrow, umbilical cord, teeth, adipose, and so on. However, though all these cells share similar cell surface markers, tri-lineage differentiation potentials, and immune-regulation (or immunomodulatory) capabilities, they are quite heterogeneous on the levels of transcriptomics, proteomics, secretomics, and epigenomics, resulting from age, tissue origin, genetic background, passage, and culture conditions, thus hampering their widespread clinical applications (Levy et al., 2020; Costa et al., 2021).

Therefore, reducing or eliminating the heterogeneity of MSCs is necessary for further paving the way for MSC-based therapies into clinical practice. In the past few decades, tremendous efforts have been made to characterize the origins and underlying mechanisms of the MSC heterogeneity, such as genetic modification (Jiang et al., 2019; Xu, Chen, et al., 2020), full chemical defined medium development (Xu, Lian, Chen, et al., 2020; Xu, Lian, Wu, et al., 2020), and subpopulation purifications (Levy et al., 2020).

In the current topic, review, minireview, and original research articles focusing on MSC heterogeneity were collected. The goal of this article collection is to address the most recent updates on understanding and minimizing MSC heterogeneity, including isolation, characterization, and comparison of MSCs from different origins; innovative strategies (gene modification, pre-treatment, and culture system optimization) to minimize the heterogeneity of MSCs; new biomarker development; and demonstration of MSC heterogeneity with single-cell omics or lineage tracing tools.

The well-written review paper, provided by Wruck et al., covers the most recent progress on MSC heterogeneity. After carefully analyzing the heterogeneity of MSCs from the transcriptome perspective, they concluded that the epigenetic alteration mainly contributes to the MSC heterogeneity. And then, the iMSCs, MSCs generated from the induced pluripotent stem cells (iPSCs), were proposed as an alternative to the purified MSCs from different tissues. The potentials of iMSCs for the clinical applications were also reviewed by Zhang et al. In addition to these two review papers, which discussed the MSC heterogeneity comprehensively, Mabuchi et al. summarized the characteristics of mouse bone marrow-derived MSCs, from the perspective of the microenvironment, cell markers, and gene expression profiles by integrating the single-cell RNA sequencing analysis; while Madhoun et al. reviewed the recent updates on adult human DPSCs (dental pulp stem cells) from the perspectives of cell origin, markers, heterogeneity, differentiation, immunomodulatory function, and also their therapeutic clinical applications. DPSCs, one type of MSC isolated from teeth, could be easily isolated from the disposable dental pulp with a high recovery rate and no ethical issues, highlighting their potential for regenerative therapies.

Although the iMSCs have many advantages compared to the primary MSCs, especially from the perspective of heterogeneity, the iMSCs are totally different from the primary MSCs. Moreover, biosafety is another critical issue that should be addressed carefully because of the potential genetic and epigenetic alterations during reprogramming. Therefore, most studies still focus on characterizing and reducing the heterogeneity of native MSCs. For example, in addition to the traditional characteristic of MSC, Burk et al. analyzed the MSC lipidomics (lipid phenotyping) for MSCs from different culture conditions and species. As a result, phosphatidylglycerol (PG) species were MSC-specific among different types of lipids. Furthermore, the MSCs seem to have a higher diversity of phospholipid species. Finally, two MSC lipid markers (PE O-36:3 and PG 40:7) were identified. Therefore, in addition to the transcriptomic and proteomic studies, the lipidomic study of MSCs would reveal more characteristics, functions, and heterogeneity of MSCs.

In addition to the new marker development, efforts also have been made to uncover novel mechanisms underlying the MSC heterogeneity. For example, Xie et al. analyzed the bone marrow-derived MSCs with single-cell RNA sequencing and showed that fatty acid pathways contribute to the heterogeneity of MSCs. In the bone marrow, MSCs support the maintenance of HSCs (hematopoietic stem cells) through secreting multiple growth factors. Moreover, they found that the butyrate significantly promoted HSC niche factor expression in MSCs, which had potential applications in supporting HSCs expansion with MSCs *in vitro*. Furthermore, Fei et al. analyzed the heterogeneity of PDLSCs (periodontal ligament stem cells, one type of MSC isolated from teeth) through single-cell clone analysis from the perspective of their osteogenic differentiation abilities. Moreover, the exosome containing PINK1/Parkin could promote osteogenesis through mitophagy.

The heterogeneity of MSC significantly hampers its clinical applications. Thus, it is essential to reduce the heterogeneity and improve the therapeutic efficacy for MSC-based therapy. One of the issues during MSC expansion *in vitro* is that cells become senescent slowly during the multiple subculturing, which deteriorates the therapeutic efficacy. Kim et al. demonstrated that CD26^+^ MSCs represent the subpopulation of MSCs with high cellular senescence characteristics. Furthermore, the CD26^−^ MSCs showed cell attachment enhancement, senescence reduction, and improved therapeutic efficacy in the mouse model of lung emphysema. CD26, also known as DPP4 (Dipeptidyl-Peptidase 4), could activate T cells through TCR and NF-κB pathways and promote cell adhesion via extracellular matrix modification. Although the underlying mechanism of CD26 modulating cell senescence needs to be addressed in the future, the idea of eliminating the senescent MSCs is vital for their expansion *in vitro*.

**FIGURE 1:**
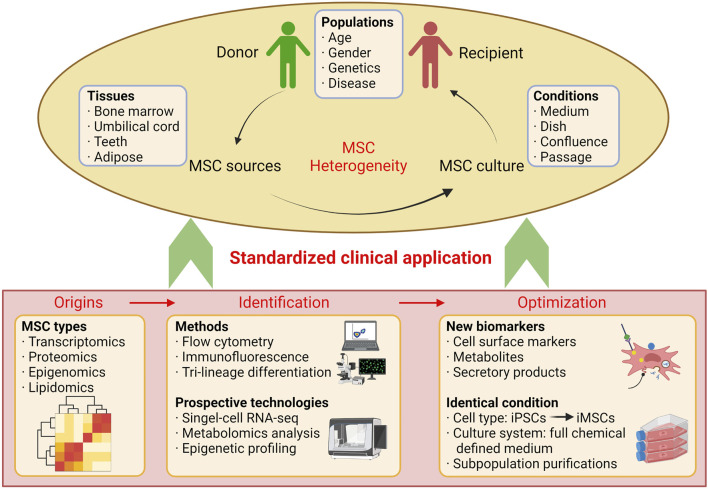
Illustration of MSC heterogeneity.

Heterogeneity is one of the cell characteristics that appeared in many cell types, including MSCs, HSCs, cancer, and iPSCs. Therefore, delineating the heterogeneity of MSCs is also essential to improve their therapeutic efficacy. This topic only collected some of those valuable manuscripts. In editing this special issue, we hope that more investigators will be attracted and inspired into this field, and more findings of the nature of MSC heterogeneity will be discovered from the perspective of basic science. Also, more convenient ways to evaluate, quantify, and minimizing the heterogeneity of MSCs are expected (Figure 1). Careful consideration should be taken before any clinical applications.
